# Targeted therapies for the treatment of soft tissue sarcoma

**DOI:** 10.3389/fonc.2023.1122508

**Published:** 2023-03-09

**Authors:** Jeffrey W. Fuchs, Brian C. Schulte, Joseph R. Fuchs, Mark Agulnik

**Affiliations:** ^1^ Department of Medicine, McGaw Medical Center of Northwestern University, Chicago, IL, United States; ^2^ Department of Medicine, University of California, San Francisco, San Francisco, CA, United States; ^3^ Medical Oncology and Therapeutics Research, City of Hope Comprehensive Cancer Center, Duarte, CA, United States

**Keywords:** soft tissue sarcoma, targeted therapy, tyrosine kinase inhibitors, clinical trial, drug therapy

## Abstract

Soft tissue sarcomas are rare malignant tumors derived from mesenchymal cells that have a high morbidity and mortality related to frequent occurrence of advanced and metastatic disease. Over the past two decades there have been significant advances in the use of targeted therapies for the treatment of soft tissue sarcoma. The ability to study various cellular markers and pathways related to sarcomagenesis has led to the creation and approval of multiple novel therapies. Herein, we describe the current landscape of targeted medications used in the management of advanced or metastatic soft tissue sarcomas, excluding GIST. We distinguish three categories: targeted therapies that have current US Food and Drug Administration (FDA) approval for treatment of soft tissue sarcoma, non-FDA approved targeted therapies, and medications in development for treatment of patients with soft tissue sarcoma.

## 1 Introduction

Soft tissue sarcomas (STS) are rare malignant tumors derived from mesenchymal cells that represent 1% of all adult malignancies in the US (1, 2). In addition to being rare in incidence, treatment of STS is complicated by the heterogenous nature of these tumors. In fact, the 2020 WHO classification of STS includes over 70 different histologic and molecular subtypes which have varied response to treatment (3). The most prevalent soft tissue sarcoma subtypes identified through registries of referral centers other than gastrointestinal stromal tumor (GIST) are liposarcoma, leiomyosarcoma, pleomorphic sarcoma, and synovial sarcoma (4, 5).

First line therapy for most advanced or metastatic STS remains anthracycline-based cytotoxic chemotherapy. For patients with neurotrophic receptor tyrosine kinase (*NTRK*) gene fusion without a known acquired resistance mutation, that are either metastatic or where surgical resection is likely to result in severe morbidity, and who have no satisfactory alternative treatments or whose cancer has progressed following treatment, TRK inhibitors are also a first-line treatment option. Advanced or metastatic STS have high morbidity and mortality with historic median progression free survival (PFS) of approximately 6 months and median overall survival of just over one year using anthracycline based chemotherapy (6–8) while more recent studies have suggested some improvement in survival with median OS of approximately 20-30 months using anthracycline-based regimens (9, 10).

While some patients with advanced or metastatic disease may benefit from local therapy of oligometastatic disease, for those who have progression on cytotoxic chemotherapy, targeted molecular therapies may be a treatment option. Over the past two decades there have been significant advances in the use of targeted molecular therapies for the treatment of STS. This has altered the landscape of STS therapy and has implications for future targeted therapies for STS which are currently in development.

In this paper we describe the current landscape of targeted therapies that are used in the management of advanced or metastatic soft tissue sarcomas, excluding GIST. We discuss medications in three categories: targeted therapies that have current US Food and Drug Administration (FDA) approval for treatment of STS, non-FDA approved targeted therapies studied in patients with STS, and medications in development for treatment of various STS histologies.

## 2 FDA approved targeted therapies for treatment of soft tissue sarcoma

### 2.1 Pazopanib

Pazopanib is an oral small molecule inhibitor of multiple tyrosine kinases including vascular endothelial growth factor receptor (VEGFR)-1, -2, and -3, platelet derived growth factor receptor (PDGFR)-α and -β, stem cell growth factor receptor (c-kit), fibroblast growth factor receptor (FGFR)-1 and -3, and colony-stimulating factor-1 receptor (c-fms) (11). Pazopanib has demonstrated utility in the treatment of all non-adipocytic STS.

In a randomized, double-blind, placebo-controlled phase III trial of 372 patients with non-adipocytic STS who had progression of disease despite standard chemotherapy, pazopanib was found to have a median progression free survival (PFS) of 4.6 months compared with 1.6 months in patients receiving placebo (hazard ratio [HR] 0.31, 95% CI 0.24-.040, *P* < 0.0001) (12). There was no statistically significant improvement in overall survival (OS) with a median OS of 12.5 months with pazopanib group versus 10.7 months with placebo. The most common adverse events (AE) were fatigue (65%), diarrhea (58%), nausea (54%), and weight loss (48%). The most common (≥ 10%) Grade ≥ 3 AE was fatigue (13%). Given the results of this study, pazopanib was FDA approved for the treatment of patients with advanced STS who have received prior chemotherapy in 2012 (13).

Pazopanib has subsequently been studied for patients with specific sarcoma histologies. In a non-comparative, randomized, open-label phase 2 trial of 72 patients with metastatic desmoid tumors, the median PFS for the 43 patients in the pazopanib group was 83.7% (95% CI 69.3 – 93.2) (14). A Phase II study of pazopanib in six patients with metastatic alveolar soft part sarcoma found one patient with partial response and five with stable disease. Median PFS was 5.5 months (95% CI 3.4-7.6 months) and the only severe toxicity noted was one case of Grade 3 diarrhea (15). In a single-arm, phase II trial of 34 patients with metastatic or unresectable typical solitary fibrous tumor, of the 31 evaluable patients, 18 (58%) had partial response 12 (39%) and had stable disease.

While typically used as a subsequent line of therapy after first-line anthracycline, pazopanib has been suggested as an initial treatment option for older adults who may not tolerate anthracycline therapy. An open-label, randomized, phase II study of pazopanib versus doxorubicin has been performed for patients age 60 years or over with progressive advanced or metastatic STS with an Eastern Cooperative Oncology Group (ECOG) performance status of 0 to 2. This study demonstrated non-inferiority of pazopanib compared with doxorubicin (16).

### 2.2 Pexidartinib

Tenosynovial giant-cell tumors (TGCTs) are benign neoplasms of joints which, while rarely metastatic, can cause significant morbidity (17, 18). TGCT cells express colony-stimulating factor-1 (CSF1) and frequently have a t(1;2) translocation of the *CSF1* gene on chromosome 1p13 to the *COL6A3* gene on chromosome 2q37 which leads to CSF1 overexpression (19–21). Therefore, CSF1/CSF1R interaction has been considered as a potential therapeutic target in the treatment of TGCT.

Pexidartinib is an orally administered, small molecule tyrosine kinase inhibitor with selective activity against colony stimulating factor 1 receptor (CSF1R) and c-kit (22). Based on its ability to act against CSF1R, pexidartinib was initially studied for the treatment of TGGT in a phase I/II dose-escalation and extension study published in 2015. For the extension group, 12 of 23 patients had partial response and 7 of 23 had stable disease (22).

Given the promising results of this dose-escalation and extension study, a randomized, phase III trial of pexidartinib versus placebo was conducted in 120 patients with advanced TGCT (23). Results of this study showed a 39% overall response rate compared to placebo (0%) (*P* < 0.001). Patients on pexidartinib also reported significantly increased range of motion (+15% with pexidartinib versus +6% with placebo, *P* = 0.0043) and significantly improved physical functioning (*P* = 0.0019) per the Patient-Reported Outcomes Measurement Information System – Physical Function scale (PROMIS). The most common AEs were hair color change (67%), fatigue (54%), aspartate aminotransferase increase (39%), nausea (38%), alanine aminotransferase increase (28%), and dysgeusia (25%). The most common (≥ 10%) Grade ≥ 3 AE were aspartate aminotransferase increase (10%) and alanine aminotransferase increase (10%).

Of note, emergence of mixed or cholestatic hepatotoxicity led to a shortened enrollment period and enrollment was halted six patients short of target. Three patients in the pexidartinib group had aminotransferase levels three or more times the upper limit of normal with total bilirubin and alkaline phosphatase two or more times the upper limit of normal indicative of mixed or cholestatic hepatoxicity. One patient required two liver dialysis procedures. However, with longer pexidartinib treatment no additional cases of mixed and cholestatic hepatoxicity occurred.

Pexidartinib was FDA approved for the treatment of adult patients with symptomatic TGCT associated with severe morbidity or functional limitations and not amenable to improvement with surgery in August 2019 (24). Given the reports of liver injury, pexidartinib has a boxed warning of hepatoxicity and is available through a Risk Evaluation and Mitigation Strategy (REMS) program.

### 2.3 Imatinib

Imatinib is an orally bioavailable multikinase inhibitor. Designed as an inhibitor of BCR-ABL, imatinib has been found to have multiple tyrosine kinase activity including against PDGFR-α, -β, and c-kit (25). In 2006 the US FDA approved imatinib for the treatment of adult patients with unresectable, recurrent and/or metastatic dermatofibrosarcoma protuberans (DFSP) which harbors t(17;22)(COL1A1;PDGFB) fusion protein in the majority of cases (26, 27).

Imatinib has been studied in two phase II trials for the treatment of locally advanced or metastatic DFSP harboring t(17;22) and found to have objective response rate approaching 50% (28). The most common (≥10%) Grade 3 AEs of imatinib in the treatment of DFSP are neutropenia (16.7%) and fatigue (16.7%).

Long-term results of a single-institution study of 31 patients with locally advanced/initially inoperable/or metastatic DFSP (including those with fibrosarcomatous transformation) treated with imatinib demonstrated a 5-year PFS of 58% and 5-year OS of 64% (29).

Finally, an updated systematic review published in 2019 showed complete response in 5.2% of patients, partial response rate of 55.2%, and stable disease in 27.6% of 152 patients treated with imatinib for locally advanced or metastatic DFSP (30).

### 2.4 Crizotinib

Crizotinib is an orally available, small molecule tyrosine kinase inhibitor of c-Met, anaplastic lymphoma kinase (ALK), and ROS1 which has FDA approval for treatment of ALK or ROS1-positive non-small cell lung cancer (31, 32). In January 2022, crizotinib was FDA approved for treatment of pediatric and adult unresectable, recurrent, or refractory ALK-positive inflammatory myofibroblastic tumors (IMT).

Crizotinib has been studied in two open-label trials, one in the pediatric population and one in the adult population. An open-label, phase I dose-escalation study of patients older than 12 months and younger than 22 years with refractory measurable or evaluable solid, CNS tumors, or anaplastic large cell lymphoma was performed (33). Seven patients were enrolled in this study with ALK-positive IMT. Of these patients, 4 had SD and 3 had PR. The most common Grade ≥ 3 AEs was decreased neutrophil count.

In a single-arm, open-label, phase Ib trial of crizotinib for adolescent and adult patients ≥ 15 years old with ALK-positive advanced malignancies other than non-small cell lung cancer, 44 patients were enrolled of which 9 had ALK-positive IMT (34). Of the 9 patients with IMT, 67% (95% CI 30-93) had response with 1 complete response and 5 partial responses. After two years, three of these patients still showed response. The most common Grade ≥ 3 AE for all patients were neutropenia 22.7%, elevated transaminases 6.8%, and vomiting 6.8%.

### 2.5 Tazemetostat

More than 90% of epithelioid sarcoma (ES) tumors lack expression of INI1/SMARCB1, an epigenetic regulator. Loss of INI1 function allows the histone methyltransferase and epigenetic modifier Enhancer of Zeste Homolog 2 (EZH2) to act as an oncogenic driver in tumor cells (35).

Tazemetostat was developed as an orally available, small molecule selective inhibitor of S-adenosyl methionine (SAM) competitive inhibitor of EZH2 (36). Tazemetostat was initially studied in a phase I trial of relapsed or refractory B-cell non-Hodgkin lymphoma and advanced solid tumors including 3 patients with INI1-negative ES (37).

In an open-label, phase II basket study of patients with INI1-negative solid tumors and synovial sarcoma treated with tazemetostat, results were published for the ES cohort. Of the 62 patients in the ES cohort, tazemetostat showed objective response in 15% of patients at data cutoff and a disease control rate of 26% at 32 weeks (38). The most commonly reported AE were fatigue (37%), nausea (35%), and cancer pain (27%). The only Grade ≥ 3 AE in more than 10% of the study population was anemia (13%). In June 2020, based on the results of this study, the FDA gave accelerated approval for the treatment of patients aged 16 or older with metastatic or locally advanced ES not eligible for complete resection (39).

### 2.6 Nanoparticle albumin*-*bound sirolimus (*nab*-sirolimus)

Perivascular epithelioid cell tumor (PEComa) is an ultra-rare type of STS with an estimated annual incidence of ≤1 per 1,000,000 population (40). PEComas often have mutations in or loss of *TSC1* or *TSC2* genes which leads to increased mammalian target of rapamycin (mTOR) activity (41, 42). It is thought that mTOR activation is a driver of cell proliferation in PEComa and mTOR has subsequently been used as a therapeutic target with mTOR inhibitors as evaluated in retrospective analyses and case series (43, 44).

Given the variable oral absorption and bioavailability of sirolimus and everolimus, intravenous nanoparticle albumin-bound (*nab*-sirolimus) has been studied in the treatment of advanced malignant PEComa. Results of a prospective, open-label, phase II registration study of 31 patients who had not previously been treated with mTOR inhibitors and were available for analysis in the efficacy arm showed an overall response rate of 39% (12 of 31; 95% CI 22 to 58) with one complete response and 11 partial responses (45). Additionally, 52% (16 of 31) of patients had stable disease. Twenty-five patients had tumor profiling. Of note, 8 of 9 (89%) patients with a *TSC2* mutation achieved a confirmed response versus 2 of 16 (13%) without *TSC2* mutation (*P <*0.001).

The most common AEs ≥ 30% were mucositis (79%), fatigue (59%), rash (56%), anemia (47%), nausea (47%), diarrhea (38%), decreased weight (38%), hyperglycemia (35%), hypertriglyceridemia (32%), hypercholesterolemia (32%), and decreased appetite (32%). The most common (≥10%) Grade 3 AEs were mucositis (18%) and anemia (12%).

When *nab-*sirolimus treatment was expanded in study for use in patients who had been treated previously with mTOR inhibitors (sirolimus, everolimus, temsirolimus, or sapanisertib), 25% (4 of 16 patients) achieved partial response and 50% had stable disease. There were no Grade ≥ 4 AEs (46). *nab-*sirolimus was FDA approved for adult patients with locally advanced unresectable or metastatic malignant PEComa in November 2021 (47).

### 2.7 Tropomyosin receptor kinase inhibitors

The NTRK genes *NTRK1*, *NTRK2*, and *NTRK3* encode tropomyosin receptor kinase (TRK) proteins known as TRKA, TRKB, and TRKC, respectively (48). While these proteins are normally involved in neuronal development, *NTRK* gene fusions have been identified in a variety of adult and pediatric tumors types (49). These gene fusions encode proteins which have constitutive TRK activity believed to be a key oncogenic driver regardless of tissue type.

Larotrectinib is an orally available, small-molecule inhibitor of all three TRK proteins and has been studied in a phase II basket study of adults and adolescents with *TRK* fusion-positive cancers (50). Seven (13%) patients had infantile fibrosarcoma and 11 (20%) of the patients in the study had “other” soft tissue sarcoma including myopericytoma (two patients), sarcoma that was not otherwise specified (two patients), peripheral-nerve sheath tumor (two patients), spindle-cell tumor (three patients), infantile myofibromatosis (one patient), and inflammatory myofibroblastic tumor of the kidney (one patient).

The overall response rate for all tumor types was 75% (95% CI, 61 - 85) as determined by independent radiology review committee. Of the 55 patients in the study, 7 patients had complete response, 34 had a partial response, and 7 had stable disease. The median time to response was 1.8 months. At 1 year, 71% of responses were ongoing and 55% of all patients remained progression-free.

The most common (>30%) AEs, regardless of attribution, were fatigue (36%), vomiting (33%), nausea (31%), dizziness (31%), and increased ALT or AST (42%). The only Grade ≥ 3 AE regardless of attribution in more than 10% of patients was anemia (11%). Larotrectinib was granted accelerated FDA approval for adult and pediatric patients with solid tumors that have *NTRK* gene fusion without a known acquired resistance mutation, that are either metastatic or where surgical resection is likely to result in severe morbidity, and who have no satisfactory alternative treatments or whose cancer has progressed following treatment in November 2018 (51).

Entrectinib is an orally available inhibitor of all three TRK proteins that has the ability to cross the blood-brain barrier. A review of two phase I (ALKA-372-001 and STARTRK-1) and one phase II (STARTRK-2) clinical trials of entrectinib for NTRK fusion-positive has been conducted (52). There were 13 patients with various types of soft tissue sarcoma included in this analysis.

At the data cutoff (May 31, 2018), the efficacy-available population of 54 adults and 12.9 months of median follow-up showed a 57% objective response including 7% complete response and 50% partial response with a median duration of response of 10 months for all tumor types. The most common (≥10%) Grade ≥ 3 AEs in patients in the NTRK fusion-positive safety population were increased weight (10%) and anemia (12%). Three serious treatment-related events occurred in the NTRK fusion-positive positive population: cognitive disorder, cerebellar ataxia, and dizziness.

In an updated analysis of 150 adults with NTRK fusion-positive tumors treated with entrectinib across 17 solid tumor types, the objective response rate was 61.3% with 16.7% complete responses (53). Thirty-two of the patients in this analysis had NTRK fusion-positive sarcomas and an objective response rate was seen in 19 (59.4%) of these patients. The median duration of response for all NTRK fusion-positive tumor types was 20 months (95% CI 13.2 – 31.1), median progression free survival was 13.8 months (95% CI 10.1 – 20.0), and median overall survival was 37.1 months (95% CI 27.2 – not estimable).

Given that entrectinib crosses the blood brain barrier, patients with CNS metastases were included in this study. In patients with investigator-assessed baseline CNS disease, objective response rate was seen in 61.3% (95% CI 42.2 – 78.2) of patients with baseline CNS metastases compared to 61.3% (95% CI 52.0 – 70.1) in patients without CNS disease.

Entrectinib has been well-tolerated among patients with the most common treatment related AEs being Grade 1/2 including dysgeusia (36.6%), diarrhea (29.8%), and weight increase (28.5%). Adverse events led to dose interruption in 32.8% of patients, dose reduction in 24.3% of patients, and discontinuation in 7.2% of patients.

The most current data on use of entrectinib for NTRK fusion-positive sarcoma was presented at the CTOS Annual Meeting in November 2022 (54). In the sarcoma efficacy population of 26 patients (2 with baseline CNS disease and 24 without baseline CNS disease), 11.5% (2 of 26) had complete response, 46.2% (12 of 26) had partial response, 15.4% (4 of 26) had stable disease. The median duration of response was 15.0 months (95% CI 4.6 – not evaluable). While both patients with baseline CNS disease had at least a partial response, only one patient had a durable response to therapy. Seventeen of thirty-seven patients in the sarcoma safety group experienced a Grade ≥ 3 AE. The most common Grade 3 treatment-related AE was increased weight in 10.8% of patients and there was one Grade 4 treatment-related AE of hyperuricemia.

Entrectinib was granted accelerated FDA approval for adults and pediatric patients 12 years of age and older with solid tumors that that have *NTRK* gene fusion without a known acquired resistance mutation, that are either metastatic or where surgical resection is likely to result in severe morbidity, and who have no satisfactory alternative treatments or whose cancer has progressed following treatment in August 2019 (55).

## 3 Non-FDA approved targeted therapies studied in patients with soft tissue sarcomas

### 3.1 Regorafenib

Regorafenib is a an orally bioavailable multikinase inhibitor of VEGFR-1, -2, and -3, tyrosine kinase with immunoglobulin and epidermal growth factor homology domain 2, and KIT (56). It is chemically similar to sorafenib with the addition of a fluorine atom in the center phenyl ring. Regorafenib has met primary endpoints in phase III trials of patients with metastatic colorectal cancer (57, 58), locally advanced, unresectable, or metastatic GIST (59), and hepatocellular carcinoma (60).

In a randomized, placebo-controlled, phase II trial of 182 patients with non-GIST STS subtypes who had progressed or were intolerant to anthracycline-based chemotherapy, compared to placebo, regorafenib was shown to extend PFS in non-adipocytic STS (61). The median PFS for patients with non-adipocytic STS was 4 months with regorafenib vs. 1 month with placebo (HR 0.36, *P <*0.0001). The most common (≥10%) AEs were asthenia (13%), hand and foot skin reaction (15%), hypertension (18%), and hypophosphatemia (12%).

An open-label, single-arm phase II trial of daily regorafenib for chemotherapy-refractory, metastatic or locally advanced unresectable angiosarcoma demonstrated an overall response rate of 17.4% (4/23) with 52% (12/23) of patients showing progression free survival for greater than 4 months (62). The most common Grade ≥ 3 adverse events were decreased lymphocyte count (26%), hypertension (19%), fatigue (16%), anemia (13%), and hyponatremia (10%). Based on these results, regorafenib has been included as a treatment for metastatic or locally advanced angiosarcoma in the NCCN guidelines (1).

### 3.2 Sorafenib

Sorafenib is an oral multikinase inhibitor which was initially developed as an inhibitor of Raf kinase. This medication has been found to have broad activity against multiple tyrosine kinases including receptors involved in angiogenesis such as VEGFR-2, -3, and PDGRF-β (63). Given its anti-angiogenic properties, sorafenib has been studied for the treatment of multiple soft-tissue sarcomas including angiosarcoma, desmoid tumor (DT), and solitary fibrous tumor. Sorafenib has been identified as a preferred treatment by the NCCN soft tissue sarcoma guidelines for treatment of DT and solitary fibrous tumor (1).

A double-blind, phase III trial of sorafenib versus matching placebo has been carried out for 87 patients with progressive, symptomatic, or recurrent DTs (64). The primary end point of the study was progression-free survival (PFS). Results of the study showed a two-year PFS of 81% (95% CI 69 – 96) in the sorafenib group and 36% (95% CI 22 – 57) in the placebo group. Results also showed objective response in 33% (95% CI 20 – 48) of the fifty patients in the sorafenib group with one patient having a complete response and 15 having partial responses. Twenty percent (7 of 35 patients) (95% CI 8 – 38) in the placebo group had objective partial response. The most common grades 1 and 2 treatment related adverse events were rash (73%), fatigue (67%), hypertension (55%), diarrhea (51%), and nausea (49%) while the most common Grade ≥ 3 adverse event was rash (14%).

### 3.3 Imatinib

As previously discussed, imatinib is an oral multikinase inhibitor which has FDA approval for the treatment of locally advanced or metastatic dermatofibrosarcoma protuberans. While not FDA approved for the treatment of locally advanced or metastatic TGCT, imatinib has shown some efficacy for use in this population. A retrospective multi-institutional study of 27 patients evaluable for response showed an overall response rate in 19% of patients with 1 complete response and 4 partial responses and 74% of patients had stable disease (65). It is thought that inhibition of CSF1R by imatinib is the mechanism underlying this response and has led to investigation of CSF1R specific inhibition with medication such as pexidartinib and vimseltinib as discussed.

### 3.4 Sunitinib

Sunitinib is an orally available tyrosine kinase inhibitor with *in vivo* activity against VEGFR-2 and PDGFR-β (66, 67). Sunitinib has been studied for the treatment of solitary fibrous tumor and alveolar soft part sarcoma (ASPS).

In a retrospective analysis of 31 patients evaluable for response treated with sunitinib for advanced solitary fibrous tumor, the best responses were 2 partial response, 16 stable disease, and 13 progressive disease (67). A <30% decrease in size of tumor was observed in three patients. The median progression-free survival was 6 months.

Sunitinib has also been studied for the treatment of ASPS in a retrospective series of nine patients with advanced, translocated ASPS and evidence of progression during the three months prior to treatment (68). The median progression-free survival was 17 months and there was partial response in 5 cases, stable disease in 3 cases, and progression in one case.

### 3.5 Lenvatinib

Lenvatinib is an orally administered tyrosine kinase inhibitor that targets VEGFR1-3, FGFR1-4, PDGFRα, c-kit, and RET (69). Lenvatinib has FDA approval for the treatment for the treatment of differentiated thyroid cancer, hepatocellular carcinoma, and as part of combination therapy in the treatment of renal cell carcinoma and endometrial carcinoma (70, 71)

Pre-clinical evidence has demonstrated activity of lenvatinib in treatment of STS (72). Additionally, phase I dose-escalation studies have shown stable disease using lenvatinib in some patients with synovial sarcoma and leiomyosarcoma (73, 74).

A phase Ib/II study of lenvatinib plus eribulin has been conducted for patients with leiomyosarcoma and LPS (75). Thirty patients enrolled in the study (21 with leiomyosarcoma, 9 with LPS). The objective response rate was 19% for the leiomyosarcoma group and 20% for the LPS group. The median PFS was 8.56 months (95% CI 4.40 – Not Reached) for both groups. The most common Grade ≥ 3 AEs included neutropenia (36.7%), hand-foot syndrome (16.7%), hypertension (13.3%), proteinuria (10%), and febrile neutropenia (10%).

A phase II pilot study evaluating the efficacy of lenvatinib plus pembrolizumab in the treatment of metastatic and/or unresectable soft tissue sarcoma is currently in recruitment (Clinictrial.gov identifier: NCT04784247).

### 3.6 Crizotinib

In addition to ALK-positive IMT as discussed above, given that ASPS is characterized by translocation between chromosomes 17 and X resulting in *ASPSCR1-TFE3* fusion gene and MET overexpression, crizotinib has been studied in the treatment of advanced or metastatic ASPS (76). A non-randomized, open-label, phase II trial of 45 assessable patients with ASPS was conducted and characterized patients as being MET+ or MET- based on the presence or absence of *TFE3* gene rearrangement (76). Among the 40 MET+ patients, one patient had partial response and 35 had stable disease. The one-year PFS was 37.5% (95% CI 22.9 – 52.1). Among the 4 MET- patients one patient had partial response and 3 had stable disease. The one-year PFS for the MET- group of patients was 50% (95% CI 5.8 – 84.5). One patient had unknown MET status and had stable disease. Grade ≥ 3 treatment related AEs were fatigue in two patients and hypotension with bradycardia, blurred vision, diarrhea, and febrile neutropenia in one patient each, respectively.

### 3.7 CDK4/6 inhibitors

Palbociclib and abemaciclib are cyclin-dependent kinase CDK4/CDK6 inhibitors which are FDA approved for the treatment of advanced breast cancer (77). Given that a high percentage of well-differentiated (WD) and de-differentiated (DD) liposarcoma (LPS) demonstrate CDK4 amplification, recent trials described below have been conducted to evaluate the utility of CDK4/6 inhibitors in the treatment of LPS.

In a non-randomized, open-label, phase II trial of 60 patients with WD and DD LPS treated with single-agent palbociclib the median PFS was 17.9 weeks (2-sided 95% CI 11.9 - 24.0 weeks) with one complete response. The primary toxicity was neutropenia (grade 3, n = 20 [33%], grade 4, n = 2 [3%]) without neutropenic fever reported (78).

Abemaciclib has also been studied in a single-arm, phase II trial of patients with DD LPS. Thirty patients were enrolled in the study and 29 included for analysis. The median PFS was 30.4 weeks (95% CI 28.9 – NE) with one partial response. The observed PFS at 12 weeks was 76% (95% CI 57-90%). Grade ≥ 3 toxicities included anemia (37%), neutropenia (20%), thrombocytopenia (17%), and diarrhea (7%) (79).

A randomized, double-blind, placebo-controlled phase III study is currently in recruitment for the study of abemaciclib in patients with advanced, recurrent, or metastatic DD LPS (Clinicaltrial.gov identifier: NCT04967521).

While CDK4/6 inhibitors have most evidence for treatment of LPS, a recent phase II study evaluated palbociclib for treatment of other types of STS and osteosarcoma with have high CDK4 expression and underexpressed CDKN2A mRNA (80). Twenty-two patients who had median of three lines of prior treatment were enrolled in the study with nine different sarcoma subtypes, including two osteosarcomas represented. The median follow-up was 10 months, the median PFS was 4.2 months (95% CI 0.9-7.4), and the median 6 months PFS was 30% (95% CI 9-51). Of the 19 evaluable patients, 11 (58%) had stable disease and 8 (42%) had progression as best response. Of note, patients with higher CDK4 expression above the median showed significantly longer median PFS and OS in the univariate analysis.

## 4 Medications in development for various soft tissue sarcoma histologies

### 4.1 γ-Secretase inhibitors

The Notch signaling pathway and dysregulation of cross-talk between the Notch and Wnt/β-catenin pathway have been implicated in multiple tumor types including DT (81). γ-secretase inhibitors (GSIs) block Notch receptor proteolysis and subsequent translocation of the Notch intracellular domain to the nucleus, preventing cell cycle progression (82).

The GSI nirogacestat (PF-03084014) was studied in an open-label, phase II trial of 17 heavily pretreated adults with recurrent, progressive DT (83). Results of this study showed a 29% (5 of 17 patients) overall response rate (all partial response) for more than two years. There were also 29% (5 of 17 patients) with stable disease who remained on study. The most common AEs were Grade 1 or 2 (95%) including diarrhea (76%) and skin disorders (71%). The only Grade ≥ 3AE was hypophosphatemia (47%).

Given these results, a randomized, double-blind, placebo-controlled phase III trial of nirogacestat versus placebo has been conducted for patients with progressing DT (84). Results were presented at the European Society of Medical Oncology in 2022. There were 142 patients in the study. Nirogacestat showed improvement in PFS compared with placebo with a HR of 0.29 (95% CI 0.15 – 0.55), overall response rate was 41% with nirogacestat versus 8% with placebo *(P*<0.001), and the median time to response was 5.6 with nirogacestat versus 11.1 months with placebo. Of the AEs, most were Grade 1 or 2 (95%) and included diarrhea (84%), nausea (54%), fatigue (51%), hypophosphatemia (42%), and maculopapular rash (32%). Of note, ovarian dysfunction occurred in 75% (27/36) of women of childbearing potential and resolved in 20 (74%) who discontinued the medication.

In addition to the GSI nirogacestat, early studies of the GSIs AL101 and AL102 have demonstrated regression of DT (85, 86). Interim results of a phase II/III open-label dose regimen finding study and randomized, double-blind, placebo-controlled study of AL102 were recently presented at the European Society of Medical Oncology (ESMO) 2022 meeting (ClinicalTrials.gov identifier NCT04871282) (87). As of February 22, 2022, 31 patients had enrolled in the phase II study. Thirty patients were still on study at time of analysis and 18 of those for more than 4 weeks. Mean age was 40 years and 74% of patients were women. The most common treatment-emergent adverse effects (TEAE) ≥ 15% for all doses were diarrhea (39%), rash (26%), nausea (19%), fatigue (19%), and stomatitis (16%). Four patients had Grade 3 AEs (two deemed study-drug related: anemia, diarrhea; two deemed unrelated: vomiting, pleural effusion). There was no significant ECG or food effects noted.

### 4.2 Anlotinib

Anlotinib is an oral small-molecule inhibitor of multiple tyrosine kinases, primarily VEGFR-2 and -3, FGFR-1-4, PDGFR-α and -β, c-Kit, and Ret (88). Anlotinib first received the National Medical Products Administration of China’s approval for use in treatment of locally advanced or metastatic non-small cell lung cancer in 2018 (89). Anlotinib has since been studied extensively in the People’s Republic of China and received approval in June 2019 for second-line treatment of clear cell sarcoma, alveolar soft part sarcoma, and other soft tissue sarcomas already treated with first-line anthracyclines (90). This approval was based in part on a phase II study of 166 soft tissue sarcoma patients who had progressive disease after anthracycline-based chemotherapy and had not previously received treatment with angiogenesis inhibitors (91). The results of this study showed twelve-week PFS in 77% of patients with alveolar soft part sarcoma, 75% of patients with synovial sarcoma, and 75% of patients with leiomyosarcoma. The most common grade 3 or higher adverse events were hypertension (4.8%), triglyceride elevation (3.6%), and pneumothorax (2.4%).

Anlotinib (AL3818) is currently being studied in the US as a phase III clinical trial for the treatment of alveolar soft part sarcoma, synovial sarcoma, and leiomyosarcoma. Known as the APROMISS trial, patients with alveolar soft part sarcoma will receive open-label anlotinib while patients with leiomyosarcoma or synovial sarcoma will receive either anlotinib (two-thirds) or dacarbazine (one-third) (Clinicaltrials.gov identifier NCT03016819). At the time of this publication this study is recruiting only patients with alveolar soft part sarcoma.

Preliminary results from the APROMISS trial have evaluated anlotinib compared to dacarbazine for second line treatment of advanced or metastatic synovial sarcoma (92). Seventy-nine patients received initial treatment and were evaluable in this study with 52 receiving anlotinib as the treatment arm and 27 receiving dacarbazine as the placebo arm. Overall PFS was 2.89 months (95% CI 2.73 – 6.87) for anlotinib compared to 1.64 months (95% CI 1.45 – 2.70) for dacarbazine. The primary endpoint was met (*P =* 0.0015) with a hazard ratio of 0.449 (95% CI 0.270 – 0.744). Grade 3 treatment related adverse events were seen in 23.1% of patients treated with anlotinib and 25.9% of patients treated with dacarbazine. The most common Grade 3 adverse events for anlotinib were diarrhea (5.8%) and hypertension (3.8%).

### 4.3 MDM2 inhibitors

The *Murine Double Minute Clone 2* (*MDM2*) gene encodes an E3 ligase that binds tumor suppressor P53, both blocking the P53 transactivation domain and targeting P53 for degradation in the proteasome (93). It is thought that inhibition of MDM2 may lead to increased concentrations of P53 and restore P53 function.

MDM2 inhibition is currently being studied in a variety of cancer types given the prevalence of P53 mutations in human cancers. Amplification of MDM2 has been specifically identified in certain cancer types including LPS. In fact, amplification of MDM2 can be useful in the diagnosis of WD LPS (94).

Milademetan, an oral inhibitor of MDM2, was studied in a phase I trial of patients with advanced, relapsed, or refractory solid tumors or lymphoma (95). This study included patients with WD and DD LPS. Fifty percent of the 107 patients in this study had WD/DD LPS. Median age was 61 years and 62% of patients had received ≥3 prior therapies. Partial response was seen in 3.8% of patients and stable disease was seen in 64.2% of patients with WD/DD LPS. The most common (>10%) grade ≥ 3 drug related adverse events in the Schedule D was thrombocytopenia (14%).

Based on these phase I results, milademetan will be studied in a phase III registration study of milademetan compared to trabectedin in patients with unresectable or metastatic DD LPS that has progressed on one or more prior systemic therapies including at least one anthracycline-based therapy (Clinicaltrial.gov identifier: NCT04979442).

In addition to milademetan, BI 907828 is another MDM2-p53 inhibitor currently under study. *In vivo* study of BI 907828 for the treatment of MDM2 amplified DD LPS showed decreased tumor size and even complete response for an *in vivo* murine model (96). Based on these pre-clinical studies, BI 907282 is currently being evaluated in a phase I dose escalation/expansion study of patients with advanced solid tumors (ClinicalTrials.gov Identifier: NCT03449381).

Preliminary results have been presented for a group of 90 patients with median two lines of prior systemic therapy (97). Forty-four of the patients in the study had advanced LPS with 28 diagnosed with DD LPS and 16 diagnosed with WD LPS. At data cut-off, 34.4% of patients had received treatment for ≥ 6 months. In the 41 evaluable patients with LPS, 24 of 27 patients with DD LPS had partial response or stable disease and 13 of 14 patients with WD LPS had partial response or stable disease. The most common Grade ≥ 3 AEs were neutropenia (23.8%), thrombocytopenia (21.4%), and anemia (11.9%).

### 4.4 Vimseltinib

As discussed above, CSF1/CSF1R interaction has been a recent target for the treatment of TGCT cells given their expression of CSF1 related to the t(1;2) translocation of the *CSF1* gene on chromosome 1p13 to the *COL6A3* gene on chromosome 2q37.

Vimseltinib is an oral, switch control tyrosine kinase inhibitor which has been specifically designed for selective and potent inhibition of CSF1R (98). Initial results from a phase I (dose escalation) and phase II (expansion) study of vimseltinib for treatment of TGCT in patients with unresectable TGCT showed evidence of objective response for 30-50% of patients (99). Updated results from the phase II expansion portion for patients treated with the recommended phase II dose (30 mg twice weekly) showed partial response or stable disease in 100% with 44% of patients in Cohort A and 49% of patients in Cohort B having partial response at a median treatment duration of 7.9 and 5.7 months, respectively (100).

A randomized, double-blind, placebo-controlled, phase III trial is currently in recruitment for study of vimseltinib for patients with unresectable TGCT (Clinicaltrial.gov identifier: NCT05059262).

### 4.5 BRD9 inhibitors

Synovial sarcoma is defined by the presence of translocation t(X;18)(p11.2;q11.2) leading to the fusion of genes SYT on Chromosome 18 and SSX on Chromosome X (101). The SS18-SSX fusion oncoprotein has been found to result in genetic transcription changes through alteration in the function of SWI/SNF or BAF complexes, leading to the development of synovial sarcoma (102). Changes in canonical BAF (cBAF) complexes driven by the SS18-SSX oncoprotein causes synovial sarcoma gene expression (103, 104). One alteration this leads to is repression of SMARCB1, a cBAF complex protein that may act in tumor suppression and is found in ~70% of synovial sarcoma samples (105).

Studies have found that disruption of the ncBAF complex in samples with loss of SMARCB1 leads to attenuation of cell proliferation in synovial sarcoma (106). One subunit of ncBAF, unique from cBAF and pBAF is the BRD9, a bromodomain-containing protein. Degradation of BRD9 inhibits synovial sarcoma tumor progression in a murine model (107). Therefore, BR9D inhibitors have been developed as a possible target for treatment of synovial sarcoma.

There are two BR9D inhibitors currently under early phase I clinical trial development for the treatment of synovial sarcoma. CFT8634 is an oral heterobifunctional degrader that bridges BRD9 with E3 ligase, causing ubiquitination and proteasomal degradation of BRD9 (108). A phase I clinical trial is currently recruiting to assess the safety and tolerability of CFT8634 in locally advanced or metastatic SMARCB1-Perturbed cancers including synovial sarcoma and SMARCB1-Null tumors who have been previous treated with at least one prior line of systemic therapy (ClinicalTrials.gov Identifier: NCT05355753).

FHD-609 is an intravenous BRD9 degrader that bridges BRD9 with cereblon (CRBN) E3 ubiquitin ligase substrate that leads to proteasomal degradation (109). A phase I, open-label, dose escalation and expansion study is currently recruiting patients to evaluate the safety, tolerability, and preliminary clinical activity of FHD-609 for patients with advanced synovial sarcoma or advanced SMARCB1-loss tumors (ClinicalTrials.gov Identifier: NCT04965753).

## 5 Discussion

Targeted therapies for treatment of locally advanced and metastatic STS have historically relied on tyrosine kinase inhibition (TKI) with pazopanib for non-adipocytic STS. Additional TKIs have been studied in STS including imatinib, regorafenib, sorafenib, sunitinib, lenvatinib, and crizotinib. These TKIs are multikinase inhibitors and thought to have activity in treatment of STS given their ability to inhibit angiogenesis and tumor growth promoting receptor tyrosine kinases.

**Figure 1:**
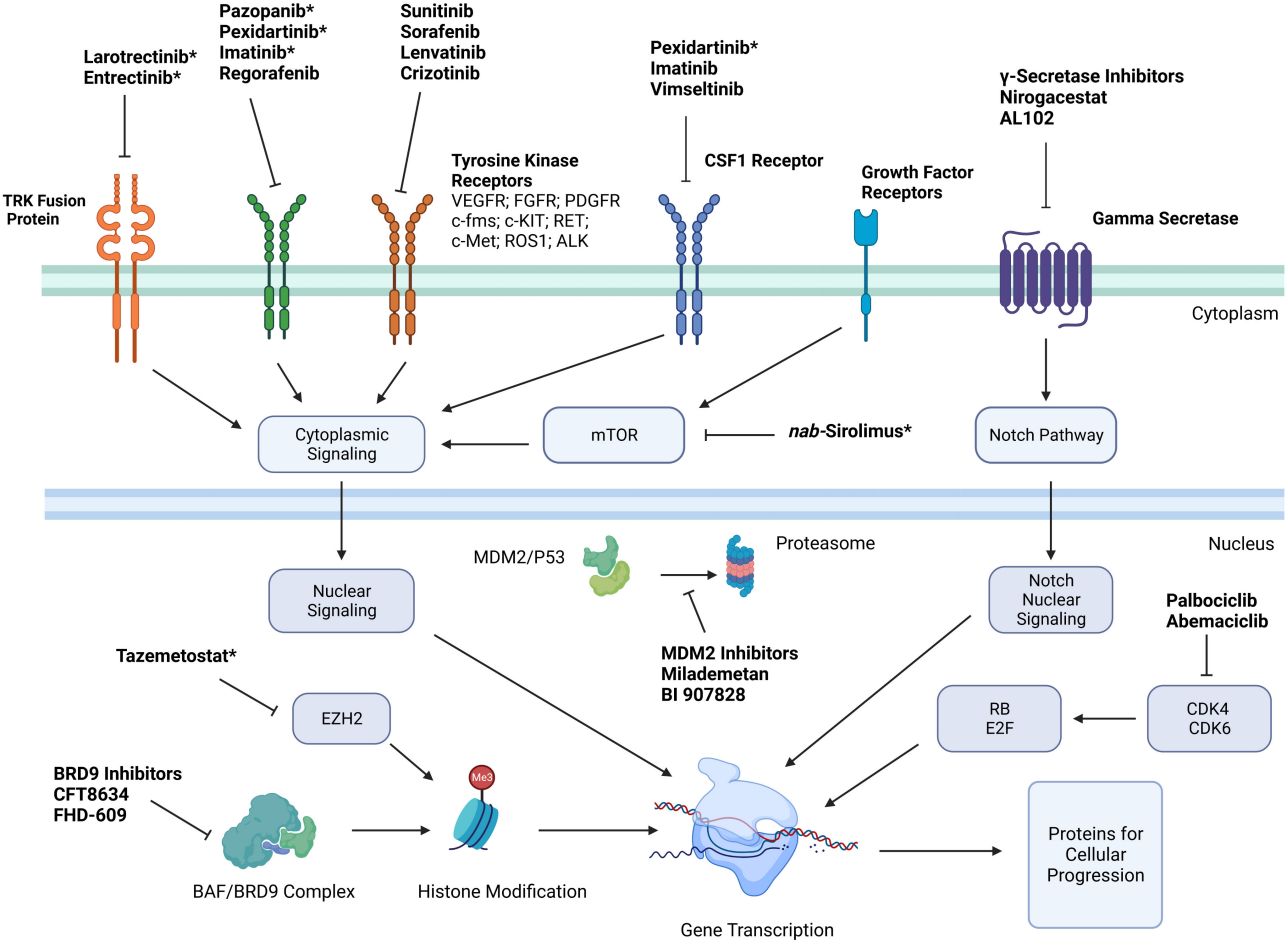
Simplified mechanisms of action of targeted therapies for treatment of soft tissue sarcoma. *Indicates that medication is FDA approved for treatment of certain soft tissue sarcoma subtypes. Key: Platelet derived growth factor receptor (PDGFR); vascular endothelial growth factor receptor (VEGFR); stem cell growth factor receptor (c-kit); Hepatocyte Growth Factor Receptor (c-Met); Anaplastic Lymphoma Kinase (ALK); cyclin dependent kinase (CDK); colony stimulating factor 1 (CSF1); enhancer of zeste homolog 2 (EZH2); retinoblastoma (RB).

With improved understanding of the cellular markers and possible driver mutations causing sarcomagenesis for different STS subtypes, multiple targeted therapies have been developed to directly inhibit these cellular processes with the hope of objective tumor response. Simplified mechanisms of action of these therapies can be seen in **Figure 1**. These therapies include FDA approved treatments with a wide variety of specific mechanisms listed in **Table 1**.

**Table 1:** FDA Approved Targeted Therapies for Treatment of Soft Tissue Sarcoma.

Medication	Mechanism of Action	Target	Sarcoma Type
Pazopanib	Tyrosine Kinase Inhibitor	VEGFR-1,-2,-3; PDGFR-α,-β; c-kit; FGFR-1,-3; c-fms	Non-adipocytic STS (12)
Pexidartinib	Tyrosine Kinase Inhibitor	CSF1R; c-kit	TGCT (23)
Imatinib	Tyrosine Kinase Inhibitor	PDGFR-β	Dermatofibrosarcoma Protuberans (28–30)
Crizotinib	Tyrosine Kinase Inhibitor	c-Met; ALK; ROS1	IMT (33, 34)
Tazemetostat	EZH2 Inhibitor	EZH2	Epithelioid Sarcoma (38)
*nab-*Sirolimus	mTOR inhibitor	mTOR Pathway	PEComa (45, 46)
Larotrectinib Entrectinib	TRK inhibitor	TRK	TRK Fusion-Positive Tumors (50, 52–54)

Vascular endothelial growth factor receptor (VEGFR); platelet derived growth factor receptor (PDGFR); stem cell growth factor receptor (c-kit); fibroblast growth factor receptor (FGFR); colony-stimulating factor-1 receptor (c-fms); tenosynovial giant cell tumor (TGCT); hepatocyte growth factor receptor (c-Met); anaplastic lymphoma kinase (ALK); inflammatory myofibroblastic tumor (IMT); mTOR (mammalian target of rapamycin); perivascular epithelioid tumor (PEComa).

Multiple medications are currently in development for the treatment of STS which are directed at known targets from previously effective therapies including anlotinib (TKI) and vimseltinib (CSF1R inhibitor) and are listed in **Table 2**. Successive generations of medications targeting known STS drivers may have high receptor affinity and decrease adverse events.

**Table 2:** Non-FDA Approved Targeted Therapies Studied in Patients with Soft Tissue Sarcoma.

Medication	Mechanism of Action	Target	Sarcoma Type
Regorafenib	Tyrosine Kinase Inhibitor	PDGFRα; VEGFR-1, -2, -3; c-kit	Non-adipocytic STS (61) Angiosarcoma (62)
Sorafenib	Tyrosine Kinase Inhibitor	Raf Kinase; VEGFR-2, -3; PDGFR-β	Desmoid Tumor (64)
Imatinib	Tyrosine Kinase Inhibitor	ABL; PDGFR; c-kit Possible CS1FR	TGCT (65)
Sunitinib	Tyrosine Kinase Inhibitor	VEGFR-2, PDGFR-β	Solitary Fibrous Tumor (67) ASPS (68)
Lenvatinib	Tyrosine Kinase Inhibitor	VEGFR1-3; FGFR1-4; PDGFRα; c-kit; RET	Leiomyosarcoma (75) LPS (75)
Crizotinib	Tyrosine Kinase Inhibitor	c-Met; ALK; ROS1	ASPS (76)
Palbociclib Abemaciclib	CDK4/6 Inhibitor	CDK 4/6	WD/DD LPS (78, 79) STS with high CDK4 expression (80)

Platelet derived growth factor receptor (PDGFR); vascular endothelial growth factor receptor (VEGFR); stem cell growth factor receptor (c-kit); hepatocyte growth factor receptor (c-Met); anaplastic lymphoma kinase (ALK); cyclin dependent kinase (CDK); tenosynovial giant cell tumor (TGCT); Alveolar Soft Part Sarcoma (ASPS); well-differentiated/dedifferentiated liposarcoma (WD/DD LPS).

Medications currently under investigation for treatment of STS with novel mechanisms of action include γ-secretase inhibitors (Notch and WNT/β-catenin pathway) for treatment of DT, MDM2 inhibitors targeting P53 for treatment of LPS given high expression of MDM2 in this STS subtype, and BRD9 inhibitors targeting ncBAF complex for treatment of synovial sarcoma and other SMARCB1-loss tumors. These medications and related clinical trials are listed in **Table 3**.

**Table 3:** Medications in Development for Treatment of Various Soft Tissue Sarcoma Histologies.

Medication	Mechanism of Action	Target	Sarcoma Type	ClinicalTrials.gov Identifier
Nirogacestat AL102	γ-Secretase Inhibitors	Notch and Wnt/β-catenin pathway	Desmoid Tumor	NCT03785964 NCT04871282
Anlotinib	Tyrosine Kinase Inhibitor	VEGFR-2,-3; FGFR-1,-4; PDGFR-α,-β; c-Kit; Ret	ASPS Synovial Sarcoma Leiomyosarcoma	NCT03016819
Milademetan BI 907828	MDM2 Inhibitor	P53	WD/DD LPS	NCT04979442 NCT03449381
Vimseltinib	Tyrosine Kinase Inhibitor	CSF1R	TGCT	NCT05059262
CFT8634 FHD-609	BRD9 Inhibitor	ncBAF Complex	Synovial Sarcoma SMARCB1-Loss Tumors	NCT05355753 NCT04965753

Vascular endothelial growth factor receptor (VEGFR); platelet derived growth factor receptor (PDGFR); fibroblast growth factor receptor (FGFR); alveolar Soft Part Sarcoma (ASPS); well-differentiated/dedifferentiated liposarcoma (WD/DD LPS); Murine Double Minute Clone 2 (MDM2); tenosynovial Giant Cell Tumor (TGCT).

Given the interest in immunotherapy for treatment of STS, future studies may seek to combine targeted therapy with immunotherapy to evaluate if there is enhancement in treatment effect and improved patient outcomes (110, 111). These studies must be mindful of adverse effects of combination immunotherapy as has been seen in previous study (112, 113).

In addition to therapies that target specific cellular and molecular mechanisms as discussed, research is also underway to identify drug delivery systems which may improve patient outcomes. Nanoparticle albumin-bound sirolimus (*nab*-sirolimus) is an example of a targeted therapy (mTOR inhibitor) which had improved therapeutic dosing with a nanoparticle drug delivery system. Future work will explore drug delivery systems with the hope to enhance the effect of chemotherapy, molecular targeted therapies, and radiation therapy while reducing toxicity (114).

## 6 Conclusion

Over the past two decades there has been significant advancement in the use of targeted therapies for the treatment of advanced and metastatic STS. These developments in targeted therapies have highlighted a key paradigm and future direction of treatment. Continuing in this vein, and building on the success of the prior years, it is easy to see that the future of treatment in sarcoma is bright. Next generation sequencing of STS in later lines will continue to improve, and with it, our ability to identify actionable targets. The promise of treatments that minimize toxicity, while maximizing on target efficacy is hard to ignore, and with the rapid pace of development, may shortly be in reach.

## Author contributions

MA conceptualized the manuscript. JWF and JRF wrote the original draft. JRF, JWF, BS, and MA were responsible for writing and editing subsequent drafts and providing final approval for the manuscript. All authors contributed to the article and approved the submitted version.

## References

[1] von Mehren M Kane JM Agulnik M Bui MM Carr-Ascher J Choy E. Soft tissue sarcoma, version 2.2022, NCCN clinical practice guidelines in oncology. J Natl Compr Canc Netw (2022) 20:815–33. doi: 10.6004/jnccn.2022.0035 PMC10186762 35830886

[2] Siegel RL Miller KD Fuchs HE Jemal A. Cancer statistics, 2022. CA Cancer J Clin (2022) 72:7–33. doi: 10.3322/caac.21708 35020204

[3] WHO Classification of Tumours Editorial Board Soft tissue and bone tumors. (2020).

[4] Coindre J-M Terrier P Guillou L Le Doussal V Collin F Ranchère D. Predictive value of grade for metastasis development in the main histologic types of adult soft tissue sarcomas. Cancer (2001) 91:1914–26. doi: 10.1002/1097-0142(20010515)91:10<1914::AID-CNCR1214>3.0.CO;2-3 11346874

[5] Zagars GK Ballo MT Pisters PWT Pollock RE Patel SR Benjamin RS. Prognostic factors for patients with localized soft-tissue sarcoma treated with conservation surgery and radiation therapy. Cancer (2003) 97:2530–43. doi: 10.1002/cncr.11365 12733153

[6] Van Glabbeke M van Oosterom AT Oosterhuis JW Mouridsen H Crowther D Somers R. Prognostic factors for the outcome of chemotherapy in advanced soft tissue sarcoma: an analysis of 2,185 patients treated with anthracycline-containing first-line regimens–a European organization for research and treatment of cancer soft tissue and bone sarcoma group study. J Clin Oncol (1999) 17:150–7. doi: 10.1200/JCO.1999.17.1.150 10458228

[7] Judson I Verweij J Gelderblom H Hartmann JT Schöffski P Blay J-Y. Doxorubicin alone versus intensified doxorubicin plus ifosfamide for first-line treatment of advanced or metastatic soft-tissue sarcoma: A randomised controlled phase 3 trial. Lancet Oncol (2014) 15:415–23. doi: 10.1016/S1470-2045(14)70063-4 24618336

[8] Ryan CW Merimsky O Agulnik M Blay J-Y Schuetze SM Tine BAV. PICASSO III: A phase III, placebo-controlled study of doxorubicin with or without palifosfamide in patients with metastatic soft tissue sarcoma. J Clin Oncol (2016) 34(32):3898–905. doi: 10.1200/JCO.2016.67.6684 27621408

[9] Tap WD Wagner AJ Schöffski P Martin-Broto J Krarup-Hansen A Ganjoo KN. Effect of doxorubicin plus olaratumab vs doxorubicin plus placebo on survival in patients with advanced soft tissue sarcomas: The ANNOUNCE randomized clinical trial. JAMA (2020) 323:1266–76. doi: 10.1001/jama.2020.1707 PMC7139275 32259228

[10] D’Ambrosio L Touati N Blay J-Y Grignani G Flippot R Czarnecka AM. Doxorubicin plus dacarbazine, doxorubicin plus ifosfamide, or doxorubicin alone as a first-line treatment for advanced leiomyosarcoma: A propensity score matching analysis from the European organization for research and treatment of cancer soft tissue and bone sarcoma group. Cancer (2020) 126:2637–47. doi: 10.1002/cncr.32795 32129883

[11] Kumar R Knick VB Rudolph SK Johnson JH Crosby RM Crouthamel M-C. Pharmacokinetic-pharmacodynamic correlation from mouse to human with pazopanib, a multikinase angiogenesis inhibitor with potent antitumor and antiangiogenic activity. Mol Cancer Ther (2007) 6:2012–21. doi: 10.1158/1535-7163.MCT-07-0193 17620431

[12] van der Graaf WTA Blay J-Y Chawla SP Kim D-W Bui-Nguyen B Casali PG. Pazopanib for metastatic soft-tissue sarcoma (PALETTE): A randomised, double-blind, placebo-controlled phase 3 trial. Lancet (2012) 379:1879–86. doi: 10.1016/S0140-6736(12)60651-5 22595799

[13] The ASCO Post. FDA Approves pazopanib for advanced soft-tissue sarcoma (2012). Available at: https://ascopost.com/issues/may-15-2012/fda-approves-pazopanib-for-advanced-soft-tissue-sarcoma/ (Accessed January 8, 2023).

[14] Toulmonde M Pulido M Ray-Coquard I Andre T Isambert N Chevreau C. Pazopanib or methotrexate–vinblastine combination chemotherapy in adult patients with progressive desmoid tumours (DESMOPAZ): A non-comparative, randomised, open-label, multicentre, phase 2 study. Lancet Oncol (2019) 20:1263–72. doi: 10.1016/S1470-2045(19)30276-1 31331699

[15] Kim M Kim TM Keam B Kim YJ Paeng JC Moon KC. A phase II trial of pazopanib in patients with metastatic alveolar soft part sarcoma. Oncologist (2019) 24:20–e29. doi: 10.1634/theoncologist.2018-0464 30254189 PMC6324645

[16] Grünwald V Karch A Schuler M Schöffski P Kopp H-G Bauer S. Randomized comparison of pazopanib and doxorubicin as first-line treatment in patients with metastatic soft tissue sarcoma age 60 years or older: Results of a German intergroup study. J Clin Oncol (2020) 38:3555–64. doi: 10.1200/JCO.20.00714 32840417

[17] Healey JH Bernthal NM van de Sande M. Management of tenosynovial giant cell tumor: A neoplastic and inflammatory disease. J Am Acad Orthop Surg Glob Res Rev (2020) 4:e20.00028. doi: 10.5435/JAAOSGlobal-D-20-00028 PMC7643913 33156160

[18] Gelhorn HL Tong S McQuarrie K Vernon C Hanlon J Maclaine G. Patient-reported symptoms of tenosynovial giant cell tumors. Clin Ther (2016) 38:778–93. doi: 10.1016/j.clinthera.2016.03.008 PMC5469507 27041409

[19] West RB Rubin BP Miller MA Subramanian S Kaygusuz G Montgomery K. A landscape effect in tenosynovial giant-cell tumor from activation of CSF1 expression by a translocation in a minority of tumor cells. Proc Natl Acad Sci U S A. (2006) 103:690–5. doi: 10.1073/pnas.0507321103 PMC1325107 16407111

[20] Cupp JS Miller MA Montgomery KD Nielsen TO O’Connell JX Huntsman D. Translocation and expression of CSF1 in pigmented villonodular synovitis, tenosynovial giant cell tumor, rheumatoid arthritis and other reactive synovitides. Am J Surg Pathol (2007) 31:970–6. doi: 10.1097/PAS.0b013e31802b86f8 17527089

[21] Möller E Mandahl N Mertens F Panagopoulos I. Molecular identification of COL6A3-CSF1 fusion transcripts in tenosynovial giant cell tumors. Genes Chromosomes Cancer (2008) 47:21–5. doi: 10.1002/gcc.20501 17918257

[22] Tap WD Wainberg ZA Anthony SP Ibrahim PN Zhang C Healey JH. Structure-guided blockade of CSF1R kinase in tenosynovial giant-cell tumor. N Engl J Med (2015) 373:428–37. doi: 10.1056/NEJMoa1411366 26222558

[23] Tap WD Gelderblom H Palmerini E Desai J Bauer S Blay J-Y. Pexidartinib versus placebo for advanced tenosynovial giant cell tumour (ENLIVEN): A randomised phase 3 trial. Lancet (2019) 394:478–87. doi: 10.1016/S0140-6736(19)30764-0 PMC6860022 31229240

[24] U.S. Food & Drug Administration. FDA Approves pexidartinib for tenosynovial giant cell tumor (2019). Available at: https://www.fda.gov/drugs/resources-information-approved-drugs/fda-approves-pexidartinib-tenosynovial-giant-cell-tumor (Accessed January 8, 2023).

[25] Waller CF. Imatinib mesylate. In: Martens UM, editor. Small molecules in oncology. Berlin, Heidelberg: Springer (2014). p. 1–25. doi: 10.1007/978-3-642-54490-3_1

[26] cancernetwork. Gleevec gains simultaneous FDA approval for five rare, life-threatening disorders (2006). Available at: https://www.cancernetwork.com/view/gleevec-gains-simultaneous-fda-approval-five-rare-life-threatening-disorders (Accessed November 1, 2022).

[27] U.S. Food & Drug Administration Search Orphan Drug Designations and Approvals: Imatinib Mesylate. (2022). Available at: https://www.accessdata.fda.gov/scripts/opdlisting/oopd/detailedIndex.cfm?cfgridkey=208805 (Accessed December 7, 2022).

[28] Rutkowski P Glabbeke MV Rankin CJ Ruka W Rubin BP Debiec-Rychter M. Imatinib mesylate in advanced dermatofibrosarcoma protuberans: Pooled analysis of two phase II clinical trials. J Clin Oncol (2010) 28:1172–9. doi: 10.1200/JCO.2009.25.7899 PMC3040044 20194851

[29] Rutkowski P Klimczak A Ługowska I Jagielska B Wągrodzki M Dębiec-Rychter M. Long-term results of treatment of advanced dermatofibrosarcoma protuberans (DFSP) with imatinib mesylate – the impact of fibrosarcomatous transformation. Eur J Surg Oncol (2017) 43:1134–41. doi: 10.1016/j.ejso.2017.03.011 28365129

[30] Navarrete-Dechent C Mori S Barker CA Dickson MA Nehal KS. Imatinib treatment for locally advanced or metastatic dermatofibrosarcoma protuberans: A systematic review. JAMA Dermatol (2019) 155:361–9. doi: 10.1001/jamadermatol.2018.4940 PMC8909640 30601909

[31] Zou HY Li Q Lee JH Arango ME McDonnell SR Yamazaki S. An orally available small-molecule inhibitor of c-met, PF-2341066, exhibits cytoreductive antitumor efficacy through antiproliferative and antiangiogenic mechanisms. Cancer Res (2007) 67:4408–17. doi: 10.1158/0008-5472.CAN-06-4443 17483355

[32] Heigener DF Reck M. Crizotinib. In: Martens UM, editor. Small molecules in oncology. recent results in cancer research. Cham: Springer International Publishing (2018). p. 57–65. doi: 10.1007/978-3-319-91442-8_4 30069759

[33] Mossé YP Lim MS Voss SD Wilner K Ruffner K Laliberte J. Safety and activity of crizotinib for paediatric patients with refractory solid tumours or anaplastic large-cell lymphoma: A children’s oncology group phase 1 consortium study. Lancet Oncol (2013) 14:472–80. doi: 10.1016/S1470-2045(13)70095-0 PMC3730818 23598171

[34] Gambacorti-Passerini C Orlov S Zhang L Braiteh F Huang H Esaki T. Long-term effects of crizotinib in ALK-positive tumors (excluding NSCLC): A phase 1b open-label study. Am J Hematol (2018) 93:607–14. doi: 10.1002/ajh.25043 PMC5947833 29352732

[35] Stacchiotti S Zuco V Tortoreto M Cominetti D Frezza AM Percio S. Comparative assessment of antitumor effects and autophagy induction as a resistance mechanism by cytotoxics and EZH2 inhibition in INI1-negative epithelioid sarcoma patient-derived xenograft. Cancers (Basel) (2019) 11:E1015. doi: 10.3390/cancers11071015 PMC6678245 31331120

[36] National Cancer Institute. NCI drug dictionary: Definition of tazemetostat hydrobromide (2011). Available at: https://www.cancer.gov/publications/dictionaries/cancer-drug/def/tazemetostat (Accessed September 27, 2022).

[37] Italiano A Soria J-C Toulmonde M Michot J-M Lucchesi C Varga A. Tazemetostat, an EZH2 inhibitor, in relapsed or refractory b-cell non-Hodgkin lymphoma and advanced solid tumours: A first-in-human, open-label, phase 1 study. Lancet Oncol (2018) 19:649–59. doi: 10.1016/S1470-2045(18)30145-1 29650362

[38] Gounder M Schöffski P Jones RL Agulnik M Cote GM Villalobos VM. Tazemetostat in advanced epithelioid sarcoma with loss of INI1/SMARCB1: An international, open-label, phase 2 basket study. Lancet Oncol (2020) 21:1423–32. doi: 10.1016/S1470-2045(20)30451-4 33035459

[39] U.S. Food & Drug Administration. FDA Approves tazemetostat for advanced epithelioid sarcoma (2020). Available at: https://www.fda.gov/drugs/resources-information-approved-drugs/fda-approves-tazemetostat-advanced-epithelioid-sarcoma (Accessed January 8, 2023).

[40] Stacchiotti S Frezza AM Blay J-Y Baldini EH Bonvalot S Bovée JVMG. Ultra-rare sarcomas: A consensus paper from the connective tissue oncology society community of experts on the incidence threshold and the list of entities. Cancer (2021) 127:2934–42. doi: 10.1002/cncr.33618 PMC8319065 33910263

[41] Martignoni G Pea M Reghellin D Zamboni G Bonetti F. PEComas: The past, the present and the future. Virchows Arch (2008) 452:119–32. doi: 10.1007/s00428-007-0509-1 PMC2234444 18080139

[42] Kenerson H Folpe AL Takayama TK Yeung RS. Activation of the mTOR pathway in sporadic angiomyolipomas and other perivascular epithelioid cell neoplasms. Hum Pathol (2007) 38:1361–71. doi: 10.1016/j.humpath.2007.01.028 PMC2722219 17521703

[43] Wagner AJ Malinowska-Kolodziej I Morgan JA Qin W Fletcher CDM Vena N. Clinical activity of mTOR inhibition with sirolimus in malignant perivascular epithelioid cell tumors: Targeting the pathogenic activation of mTORC1 in tumors. J Clin Oncol (2010) 28:835–40. doi: 10.1200/JCO.2009.25.2981 PMC4810029 20048174

[44] Sanfilippo R Jones RL Blay J-Y Le Cesne A Provenzano S Antoniou G. Role of chemotherapy, VEGFR inhibitors, and mTOR inhibitors in advanced perivascular epithelioid cell tumors (PEComas). Clin Cancer Res (2019) 25:5295–300. doi: 10.1158/1078-0432.CCR-19-0288 31217199

[45] Wagner AJ Ravi V Riedel RF Ganjoo K Van Tine BA Chugh R. Nab-sirolimus for patients with malignant perivascular epithelioid cell tumors. J Clin Oncol (2021) 39:3660–70. doi: 10.1200/JCO.21.01728 PMC8601264 34637337

[46] Dickson MA Ravi V Riedel RF Ganjoo KN Van Tine BA Chugh R. Nab-sirolimus for patients with advanced malignant PEComa with or without prior mTOR inhibitors: Biomarker results from AMPECT and an expanded access program. J Clin Oncol (2022) 40:11574–4. doi: 10.1200/JCO.2022.40.16_suppl.11574

[47] U.S. Food & Drug Administration. FDA Approves sirolimus protein-bound particles for malignant perivascular epithelioid cell tumor (2021). Available at: https://www.fda.gov/drugs/resources-information-approved-drugs/fda-approves-sirolimus-protein-bound-particles-malignant-perivascular-epithelioid-cell-tumor (Accessed January 8, 2023).

[48] Cocco E Scaltriti M Drilon A. NTRK fusion-positive cancers and TRK inhibitor therapy. Nat Rev Clin Oncol (2018) 15:731–47. doi: 10.1038/s41571-018-0113-0 PMC6419506 30333516

[49] Stransky N Cerami E Schalm S Kim JL Lengauer C. The landscape of kinase fusions in cancer. Nat Commun (2014) 5:4846. doi: 10.1038/ncomms5846 25204415 PMC4175590

[50] Drilon A Laetsch TW Kummar S DuBois SG Lassen UN Demetri GD. Efficacy of larotrectinib in TRK fusion–positive cancers in adults and children. N Engl J Med (2018) 378:731–9. doi: 10.1056/NEJMoa1714448 PMC5857389 29466156

[51] U.S. Food & Drug Administration. FDA Approves larotrectinib for solid tumors with NTRK gene fusions (2019). Available at: https://www.fda.gov/drugs/fda-approves-larotrectinib-solid-tumors-ntrk-gene-fusions (Accessed January 8, 2023).

[52] Doebele RC Drilon A Paz-Ares L Siena S Shaw AT Farago AF. Entrectinib in patients with advanced or metastatic NTRK fusion-positive solid tumours: integrated analysis of three phase 1–2 trials. Lancet Oncol (2020) 21:271–82. doi: 10.1016/S1470-2045(19)30691-6 PMC7461630 31838007

[53] Krzakowski MJ Lu S Cousin S Smit EF Springfeld C Goto K. Updated analysis of the efficacy and safety of entrectinib in patients (pts) with locally advanced/metastatic NTRK fusion-positive (NTRK-fp) solid tumors. J Clin Oncol (2022) 40:3099–9. doi: 10.1200/JCO.2022.40.16_suppl.3099

[54] Loong H Medina L Tinoco G Garrido-Laguna I Goto K Steuer C. Updated efficacy and safety of entrectinib in patients with NTRK fusion-positive tumors, in: 2022 Connective Tissue Oncology Society Annual Meeting, Vancouver, Canada, 2022 Nov 17. Available at: https://medically.gene.com/global/en/unrestricted/oncology/CTOS-2022/ctos-2022-presentation-loong-updated-efficacy-and-safet.html.

[55] U.S. Food & Drug Administration. FDA Approves entrectinib for NTRK solid tumors and ROS-1 NSCLC (2019). Available at: https://www.fda.gov/drugs/resources-information-approved-drugs/fda-approves-entrectinib-ntrk-solid-tumors-and-ros-1-nsclc (Accessed January 8, 2023).

[56] Wilhelm SM Dumas J Adnane L Lynch M Carter CA Schütz G. Regorafenib (BAY 73-4506): A new oral multikinase inhibitor of angiogenic, stromal and oncogenic receptor tyrosine kinases with potent preclinical antitumor activity. Int J Cancer (2011) 129:245–55. doi: 10.1002/ijc.25864 21170960

[57] Grothey A Van Cutsem E Sobrero A Siena S Falcone A Ychou M. Regorafenib monotherapy for previously treated metastatic colorectal cancer (CORRECT): An international, multicentre, randomised, placebo-controlled, phase 3 trial. Lancet (2013) 381:303–12. doi: 10.1016/S0140-6736(12)61900-X 23177514

[58] Li J Qin S Xu R Yau TCC Ma B Pan H. Regorafenib plus best supportive care versus placebo plus best supportive care in Asian patients with previously treated metastatic colorectal cancer (CONCUR): A randomised, double-blind, placebo-controlled, phase 3 trial. Lancet Oncol (2015) 16:619–29. doi: 10.1016/S1470-2045(15)70156-7 25981818

[59] Demetri GD Reichardt P Kang Y-K Blay J-Y Rutkowski P Gelderblom H. Efficacy and safety of regorafenib for advanced gastrointestinal stromal tumours after failure of imatinib and sunitinib (GRID): An international, multicentre, randomised, placebo-controlled, phase 3 trial. Lancet (2013) 381:295–302. doi: 10.1016/S0140-6736(12)61857-1 23177515 PMC3819942

[60] Bruix J Qin S Merle P Granito A Huang Y-H Bodoky G. Regorafenib for patients with hepatocellular carcinoma who progressed on sorafenib treatment (RESORCE): A randomised, double-blind, placebo-controlled, phase 3 trial. Lancet (2017) 389:56–66. doi: 10.1016/S0140-6736(16)32453-9 27932229

[61] Mir O Brodowicz T Italiano A Wallet J Blay J-Y Bertucci F. Safety and efficacy of regorafenib in patients with advanced soft tissue sarcoma (REGOSARC): A randomised, double-blind, placebo-controlled, phase 2 trial. Lancet Oncol (2016) 17:1732–42. doi: 10.1016/S1470-2045(16)30507-1 27751846

[62] Agulnik M Schulte B Robinson S Hirbe AC Kozak K Chawla SP. An open-label single-arm phase II study of regorafenib for the treatment of angiosarcoma. Eur J Cancer (2021) 154:201–8. doi: 10.1016/j.ejca.2021.06.027 34284255

[63] Hahn O Stadler W. Sorafenib. Curr Opin Oncol (2006) 18:615–21. doi: 10.1097/01.cco.0000245316.82391.52 16988583

[64] Gounder MM Mahoney MR Van Tine BA Ravi V Attia S Deshpande HA. Sorafenib for advanced and refractory desmoid tumors. N Engl J Med (2018) 379:2417–28. doi: 10.1056/NEJMoa1805052 PMC6447029 30575484

[65] Cassier PA Gelderblom H Stacchiotti S Thomas D Maki RG Kroep JR. Efficacy of imatinib mesylate for the treatment of locally advanced and/or metastatic tenosynovial giant cell tumor/pigmented villonodular synovitis. Cancer (2012) 118:1649–55. doi: 10.1002/cncr.26409 21823110

[66] Mendel DB Laird AD Xin X Louie SG Christensen JG Li G. *In vivo* antitumor activity of SU11248, a novel tyrosine kinase inhibitor targeting vascular endothelial growth factor and platelet-derived growth factor receptors: Determination of a pharmacokinetic/pharmacodynamic relationship. Clin Cancer Res (2003) 9:327–37. 12538485

[67] Stacchiotti S Negri T Libertini M Palassini E Marrari A Troia BD. Sunitinib malate in solitary fibrous tumor (SFT). Ann Oncol (2012) 23:3171–9. doi: 10.1093/annonc/mds143 22711763

[68] Stacchiotti S Negri T Zaffaroni N Palassini E Morosi C Brich S. Sunitinib in advanced alveolar soft part sarcoma: Evidence of a direct antitumor effect. Ann Oncol (2011) 22:1682–90. doi: 10.1093/annonc/mdq644 21242589

[69] Yamamoto Y Matsui J Matsushima T Obaishi H Miyazaki K Nakamura K. Lenvatinib, an angiogenesis inhibitor targeting VEGFR/FGFR, shows broad antitumor activity in human tumor xenograft models associated with microvessel density and pericyte coverage. Vasc Cell (2014) 6:18. doi: 10.1186/2045-824X-6-18 25197551 PMC4156793

[70] Hao Z Wang P. Lenvatinib in management of solid tumors. Oncologist (2020) 25:e302–10. doi: 10.1634/theoncologist.2019-0407 PMC7011622 32043789

[71] U.S. Food & Drug Administration. FDA Grants regular approval to pembrolizumab and lenvatinib for advanced endometrial carcinoma (2022). Available at: https://www.fda.gov/drugs/resources-information-approved-drugs/fda-grants-regular-approval-pembrolizumab-and-lenvatinib-advanced-endometrial-carcinoma (Accessed December 20, 2022).

[72] De Vita A Recine F Miserocchi G Pieri F Spadazzi C Cocchi C. The potential role of the extracellular matrix in the activity of trabectedin in UPS and l-sarcoma: evidences from a patient-derived primary culture case series in tridimensional and zebrafish models. J Exp Clin Cancer Res (2021) 40:165. doi: 10.1186/s13046-021-01963-1 33975637 PMC8111914

[73] Hong DS Kurzrock R Wheler JJ Naing A Falchook GS Fu S. Phase I dose-escalation study of the multikinase inhibitor lenvatinib in patients with advanced solid tumors and in an expanded cohort of patients with melanoma. Clin Cancer Res (2015) 21:4801–10. doi: 10.1158/1078-0432.CCR-14-3063 PMC4840931 26169970

[74] Nakamichi S Nokihara H Yamamoto N Yamada Y Honda K Tamura Y. A phase 1 study of lenvatinib, multiple receptor tyrosine kinase inhibitor, in Japanese patients with advanced solid tumors. Cancer Chemother Pharmacol (2015) 76:1153–61. doi: 10.1007/s00280-015-2899-0 PMC4648947 26530955

[75] Chen TW-W Hsu C-L Hong R-L Lee J-C Chang K Yu C-W. A single-arm phase Ib/II study of lenvatinib plus eribulin in advanced liposarcoma and leiomyosarcoma. Clin Cancer Res (2022) 28:5058–65. doi: 10.1158/1078-0432.CCR-22-2092 36129471

[76] Schoffski P Wozniak A Kasper B Aamdal S Leahy MG Rutkowski P. Activity and safety of crizotinib in patients with advanced, metastatic alveolar soft part sarcoma (ASPS) with rearrangement of TFE3: European organization for research and treatment of cancer (EORTC) phase 2 trial 90101 CREATE. J Clin Oncol (2018) 36:11540–0. doi: 10.1200/JCO.2018.36.15_suppl.11540 29216400

[77] Spring LM Wander SA Zangardi M Bardia A. CDK 4/6 inhibitors in breast cancer: Current controversies and future directions. Curr Oncol Rep (2019) 21:25. doi: 10.1007/s11912-019-0769-3 30806829 PMC6573012

[78] Dickson MA Schwartz GK Keohan ML D’Angelo SP Gounder MM Chi P. Progression-free survival among patients with well-differentiated or dedifferentiated liposarcoma treated with CDK4 inhibitor palbociclib: A phase 2 clinical trial. JAMA Oncol (2016) 2:937–40. doi: 10.1001/jamaoncol.2016.0264 PMC4991028 27124835

[79] Dickson MA Koff A D’Angelo SP Gounder MM Keohan ML Kelly CM. Phase 2 study of the CDK4 inhibitor abemaciclib in dedifferentiated liposarcoma. J Clin Oncol (2019) 37:11004–4. doi: 10.1200/JCO.2019.37.15_suppl.11004

[80] Martin Broto J Martinez Garcia J Moura DS Redondo A Gutierrez A Lopez-Pousa A. Phase II trial of palbociclib in advanced sarcoma overexpressing CDK4 gene excluding dedifferentiated liposarcoma (DD LPS): A study from the Spanish group for research on sarcoma (GEIS). J Clin Oncol (2022) 40:11511–1. doi: 10.1200/JCO.2022.40.16_suppl.11511

[81] Shang H Braggio D Lee Y-J Al Sannaa GA Creighton CJ Bolshakov S. Targeting the notch pathway: A potential therapeutic approach for desmoid tumors. Cancer (2015) 121:4088–96. doi: 10.1002/cncr.29564 PMC4635059 26349011

[82] Riedel RF Agulnik M. Evolving strategies for management of desmoid tumor. Cancer (2022) 128:3027–40. doi: 10.1002/cncr.34332 PMC9546183 35670122

[83] Kummar S O’Sullivan Coyne G Do KT Turkbey B Meltzer PS Polley E. Clinical activity of the γ-secretase inhibitor PF-03084014 in adults with desmoid tumors (Aggressive fibromatosis). J Clin Oncol (2017) 35:1561–9. doi: 10.1200/JCO.2016.71.1994 PMC5455706 28350521

[84] Kasper B Ratan R Alcindor T Schoeffski P van der Graaf WTA Wilky BA. LBA2 DeFi: A phase III, randomized controlled trial of nirogacestat versus placebo for progressing desmoid tumors (DT). Ann Oncol (2022) 33:S1435–6. doi: 10.1016/annonc/annonc1089

[85] El-Khoueiry AB Desai J Iyer S Gadgeel S Ramalingam S Horn L. A phase I study of AL101, a pan-NOTCH inhibitor, in patients (pts) with locally advanced or metastatic solid tumors. J Clin Oncol (2018) 36:S2515–2515. doi: 10.1200/JCO.2018.36.15_suppl.2515

[86] Chan D Kaplan J Gordon G Desai J. Activity of the gamma secretase inhibitor AL101 in desmoid tumors: A case report of 2 adult cases. Curr Oncol (2021) 28:3659–67. doi: 10.3390/curroncol28050312 PMC8482204 34590610

[87] Gounder MM Jones RL Chugh R Agulnik M Singh A Tine BAV. 1488MO initial results of phase II/III trial of AL102 for treatment of desmoid tumors (DT). Ann Oncol (2022) 33:S1227–8. doi: 10.1016/j.annonc.2022.07.1591

[88] Sun Y Niu W Du F Du C Li S Wang J. Safety, pharmacokinetics, and antitumor properties of anlotinib, an oral multi-target tyrosine kinase inhibitor, in patients with advanced refractory solid tumors. J Hematol Oncol (2016) 9:1–9. doi: 10.1186/s13045-016-0332-8 27716285 PMC5051080

[89] Syed YY. Anlotinib: First global approval. Drugs (2018) 78:1057–62. doi: 10.1007/s40265-018-0939-x 29943374

[90] Li S. Anlotinib: A novel targeted drug for bone and soft tissue sarcoma. Front Oncol (2021) 11:664853. doi: 10.3389/fonc.2021.664853 34094958 PMC8173120

[91] Chi Y Fang Z Hong X Yao Y Sun P Wang G. Safety and efficacy of anlotinib, a multikinase angiogenesis inhibitor, in patients with refractory metastatic soft-tissue sarcoma. Clin Cancer Res (2018) 24:5233–8. doi: 10.1158/1078-0432.CCR-17-3766 29895706

[92] Van Tine BA Chawla SP Trent JC Wilky BA Chugh R Chmielowski B. A phase III study (APROMISS) of AL3818 (Catequentinib, anlotinib) hydrochloride monotherapy in subjects with metastatic or advanced synovial sarcoma. J Clin Oncol (2021) 39:11505–5. doi: 10.1200/JCO.2021.39.15_suppl.11505

[93] Vassilev LT. MDM2 inhibitors for cancer therapy. Trends Mol Med (2007) 13:23–31. doi: 10.1016/j.molmed.2006.11.002 17126603

[94] Weaver J Rao P Goldblum JR Joyce MJ Turner SL Lazar AJ. Can MDM2 analytical tests performed on core needle biopsy be relied upon to diagnose well-differentiated liposarcoma? Mod Pathol (2010) 23:1301–6. doi: 10.1038/modpathol.2010.106 20495536

[95] Gounder MM Bauer TM Schwartz GK LoRusso P Kumar P Kato K. Milademetan, an oral MDM2 inhibitor, in well-differentiated/ dedifferentiated liposarcoma: results from a phase 1 study in patients with solid tumors or lymphomas. Eur J Cancer (2020) 138:S3–4. doi: 10.1016/S0959-8049(20)31080-7

[96] Cornillie J Wozniak A Li H Gebreyohannes YK Wellens J Hompes D. Anti-tumor activity of the MDM2-TP53 inhibitor BI-907828 in dedifferentiated liposarcoma patient-derived xenograft models harboring MDM2 amplification. Clin Transl Oncol (2020) 22:546–54. doi: 10.1007/s12094-019-02158-z 31201607

[97] Gounder MM Yamamoto N Patel MR Bauer TM Schöffski P Grempler R. A phase Ia/Ib, dose-escalation/expansion study of the MDM2–p53 antagonist BI 907828 in patients with solid tumors, including advanced/metastatic liposarcoma (LPS). J Clin Oncol (2022) 40:3004–4. doi: 10.1200/JCO.2022.40.16_suppl.3004

[98] Smith BD Kaufman MD Wise SC Ahn YM Caldwell TM Leary CB. Vimseltinib: A precision CSF1R therapy for tenosynovial giant cell tumors and diseases promoted by macrophages. Mol Cancer Ther (2021) 20:2098–109. doi: 10.1158/1535-7163.MCT-21-0361 PMC9398179 34433663

[99] Gelderblom H Razak ARA Sánchez-Gastaldo A Rutkowski P Wilky B Wagner A. 1821P safety and preliminary efficacy of vimseltinib in tenosynovial giant cell tumor (TGCT). Ann Oncol (2021) 32:S1233–4. doi: 10.1016/j.annonc.2021.08.275

[100] Blay J-Y Gelderblom H Rutkowski P Wagner A de Sande Mv Gonzalez AF. 1509P efficacy and safety of vimseltinib in tenosynovial giant cell tumour (TGCT): Phase II expansion. Ann Oncol (2022) 33:S1236–7. doi: 10.1016/j.annonc.2022.07.1612

[101] Sun B Sun Y Wang J Zhao X Zhang S Liu Y. The diagnostic value of SYT-SSX detected by reverse transcriptase–polymerase chain reaction (RT-PCR) and fluorescence *in situ* hybridization (FISH) for synovial sarcoma: A review and prospective study of 255 cases. Cancer Sci (2008) 99:1355–61. doi: 10.1111/j.1349-7006.2008.00830.x PMC11160015 18460022

[102] Stacchiotti S Van Tine BA. Synovial sarcoma: Current concepts and future perspectives. J Clin Ocol (2018) 36:180–7. doi: 10.1200/JCO.2017.75.1941 29220290

[103] McBride MJ Pulice JL Beird HC Ingram DR D’Avino AR Shern JF. The SS18-SSX fusion oncoprotein hijacks BAF complex targeting and function to drive synovial sarcoma. Cancer Cell (2018) 33:1128–1141.e7. doi: 10.1016/j.ccell.2018.05.002 29861296 PMC6791822

[104] Kadoch C Crabtree GR. Reversible disruption of mSWI/SNF (BAF) complexes by the SS18-SSX oncogenic fusion in synovial sarcoma. Cell (2013) 153:71–85. doi: 10.1016/j.cell.2013.02.036 23540691 PMC3655887

[105] Kohashi K Oda Y Yamamoto H Tamiya S Matono H Iwamoto Y. Reduced expression of SMARCB1/INI1 protein in synovial sarcoma. Mod Pathol (2010) 23:981–90. doi: 10.1038/modpathol.2010.71 20305614

[106] Michel BC D’Avino AR Cassel SH Mashtalir N McKenzie ZM McBride MJ. A non-canonical SWI/SNF complex is a synthetic lethal target in cancers driven by BAF complex perturbation. Nat Cell Biol (2018) 20:1410–20. doi: 10.1038/s41556-018-0221-1 PMC6698386 30397315

[107] Brien GL Remillard D Shi J Hemming ML Chabon J Wynne K. Targeted degradation of BRD9 reverses oncogenic gene expression in synovial sarcoma. Elife (2018) 7:e41305. doi: 10.7554/eLife.41305 30431433 PMC6277197

[108] Jackson KL Agafonov RV Carlson MW Chaturvedi P Cocozziello D Cole K. Abstract ND09: The discovery and characterization of CFT8634: A potent and selective degrader of BRD9 for the treatment of SMARCB1-perturbed cancers. Cancer Res (2022) 82:ND09. doi: 10.1158/1538-7445.AM2022-ND09

[109] Collins M Wan J Garbitt-Amaral V Antonakos B Wu HJ Xu C. Preclinical validation of target engagement assay and investigation of mechanistic impacts of FHD-609, a clinical-stage BD9 degrader being developed for the treatment of synovial sarcoma, in: 2022 Connective Tissue Oncology Society Annual Meeting, Vancouver, Canada, 2022 Nov 17.

[110] Pollack SM Redman MW Baker KK Wagner MJ Schroeder BA Loggers ET. Assessment of doxorubicin and pembrolizumab in patients with advanced anthracycline-naive sarcoma: A phase 1/2 nonrandomized clinical trial. JAMA Oncol (2020) 6:1778–82. doi: 10.1001/jamaoncol.2020.3689 PMC7489365 32910151

[111] Ayodele O Razak ARA. Immunotherapy in soft-tissue sarcoma. Curr Oncol (2020) 27:17–23. doi: 10.3747/co.27.5407 32174754 PMC7050043

[112] Chowdhury S Infante JR Hawkins R Voss MH Perini R Arkenau T. A phase I/II study to assess the safety and efficacy of pazopanib and pembrolizumab combination therapy in patients with advanced renal cell carcinoma. Clin Genitourin Cancer (2021) 19:434–46. doi: 10.1016/j.clgc.2021.04.007 PMC9494291 34006498

[113] Motzer R Alekseev B Rha S-Y Porta C Eto M Powles T. Lenvatinib plus pembrolizumab or everolimus for advanced renal cell carcinoma. N Engl J Med (2021) 384:1289–300. doi: 10.1056/NEJMoa2035716 33616314

[114] Mercatali L Vanni S Miserocchi G Liverani C Spadazzi C Cocchi C. The emerging role of cancer nanotechnology in the panorama of sarcoma. Front Bioeng Biotechnol (2022) 10:953555. doi: 10.3389/fbioe.2022.953555 36324885 PMC9618700

